# Three-spined sticklebacks recognize familiar individuals by facial recognition

**DOI:** 10.1098/rsos.241888

**Published:** 2025-07-16

**Authors:** Shumpei Sogawa, Izumi Inoue, Satoshi Awata, Koki Ikeya, Kento Kawasaka, Masanori Kohda

**Affiliations:** ^1^ Department of Biology and Geosciences, Osaka City University, Osaka, Japan; ^2^ Department of Biology, Osaka Metropolitan University, Osaka, Japan; ^3^ World Freshwater Aquarium Aquatotto Gifu, Gifu, Japan; ^4^ Marine Biological Station, Niigata University Sado Island Center for Ecological Sustainability, Sado, Niigata, Japan

**Keywords:** three-spined stickleback, individual recognition, face recognition, class-level recognition, dear enemy, innate releasing mechanisms

## Abstract

Social vertebrates often recognize familiar individuals through facial recognition, a basal cognitive ability through which animals establish stable sociality, including territoriality. The three-spined stickleback (*Gasterosteus aculeatus*), a model species for behavioural studies, is territorial and its ability to visually recognize familiar individuals remains unclear. Herein, we report that this species has individual-specific facial features and recognizes familiar individuals by facial recognition. Territorial neighbours of the same sex established a ‘dear enemy relationship’ with each other. These focal fish were exposed to composite photographic models of four combinations of faces and bodies of familiar neighbours and unknown strangers of the same sex. Focal fish of both sexes attacked photographs of strangers (stranger-face/stranger-body) more frequently than familiar neighbours (neighbour-face/neighbour-body). Furthermore, they attacked composite photographs of the stranger-face/neighbour-body more frequently (similar to the stranger model) but less frequently attacked photographs of the neighbour-face/stranger-body (similar to the neighbour model). These results suggest that the three-spined stickleback exclusively distinguishes familiar neighbours from unknown fish via facial recognition. The aggressiveness of males was independent of the presence of a red nuptial colour in the photographs. Our findings suggest that this fish controls its aggressiveness against opponent conspecifics in the context of social relationships independent of the red area.

## 1. Introduction

The three-spined stickleback (*Gasterosteus aculeatus*), first used in studies of aggressive behaviours by Tinbergen in the 1930s [1–4], is an important model species for animal behavioural studies (e.g. [5–7]). During the breeding season, males establish breeding territories and attack the intruding conspecific males. Tinbergen interpreted the observations of a series of classic experiments as indicating that the aggressive behaviour of the three-spined stickleback is instinctively triggered by the male’s nuptial red colour on the belly, which functions as a sign-stimulus [4]. Until now this interpretation has been widely accepted; in fact, it is still included in textbooks of general biology and animal psychology as a typical example of a fixed action pattern evoked by sign-stimulus based on ‘hard-wired’ innate releasing mechanisms (IRM; e.g. [8,9]). However, several groups have suggested that the concept of a sign-stimulus is oversimplified because the red nuptial colour does not always trigger aggression, and aggression does not always require the red sign-stimulus (e.g. [10–12]).

Indeed, reciprocal altruism has been documented in three-spined sticklebacks [13,14]; nevertheless, IRM is difficult to explain this reciprocity. To maintain this sophisticated social relationship using a tit-for-tat strategy, it is predicted that this fish has various cognitive abilities, such as familiar recognition (a type of class-level recognition) and strong memory. These cognitive abilities (reciprocal altruism) and IRM seem to be mutually exclusive in explaining the same behaviour. Recently, an associative learning study yielded no evidence supporting familiar recognition abilities in three-spined sticklebacks [15]. The authors claim that this altruistic behaviour can be explained by the concept of shame/border individuality and not by individual recognition, in the context of Morgan’s Canon. Nevertheless, it is possible that in their study, the three-spined sticklebacks did have the ability of familiar recognition but might have simply failed in associative learning. Therefore, the processes employed by this animal model must be considered. Here, we examined their familiarity recognition ability from a different angle in an experiment that considered the ecological characteristics of territorial defence.

Many species of vertebrates from various taxa, including fish, have breeding territories (e.g. [16]) that are often adjacent to and surrounded by the territories of several conspecific neighbours. In mammals and birds, when a territory boundary is established between neighbours, it is rarely crossed, and they become tolerant of each other (e.g. [17,18]). This type of social relationship, called a ‘dear enemy relationship’ [19,20], has been documented in territorial cichlids and guppies [21–27].

**Figure 1.**
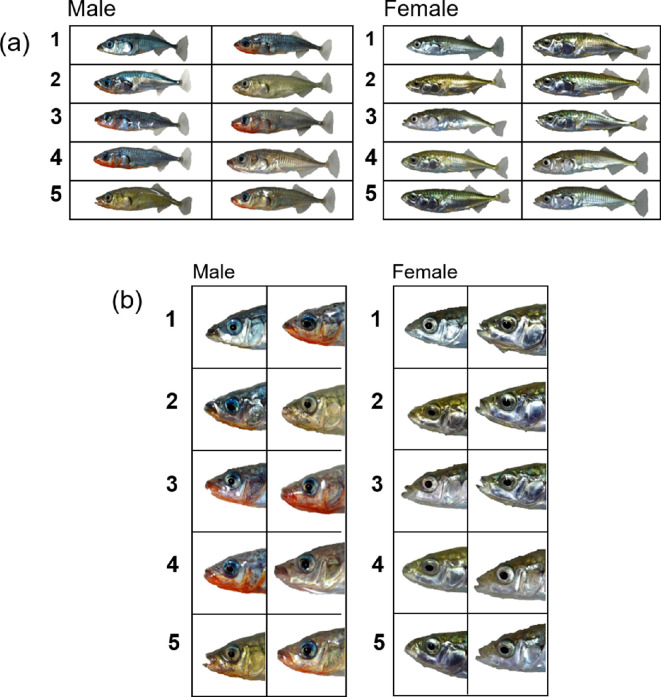
Photographs of three-spined sticklebacks. The whole body (a) and their faces (b) of 10 males and 10 females used in the present study. Pairs 1–5 are fish of both sexes used to establish dear enemy relationships (see text).

It has been suggested that such dear enemy relationships between neighbours occur in territorial three-spined sticklebacks [28]. However, their territorial interactions, including this relationship, have not been studied in detail. Analysis of these relationships provides an opportunity to study familiar recognition [23–26]. Cichlid fish that show dear enemy relationships with territorial neighbours visually discriminate familiar neighbours from strangers and can even distinguish between familiar neighbours (i.e. individual recognition) by recognizing individual-specific facial features [23–27,29,30]. Three-spined sticklebacks also has individual-specific facial colour morph characteristics (figure 1). Therefore, if territorial three-spined sticklebacks have dear enemy relationships and distinguish familiar neighbours from strangers depending on their individual-specific face colour morphs, this fish species may have an individual recognition ability similar to that of cichlids.

In the present study, we quantitatively examined whether territorial three-spined sticklebacks can establish dear enemy relationships with their conspecific neighbours, including females that are also potentially territorial under certain conditions [29], and whether this fish species can recognize and distinguish familiar neighbours from strangers based on individual-specific facial features. Familiar recognition is a cognitive ability essential for maintaining stable social relationships, including territoriality, dominance order and stable sexual pairs [31]. The process of familiar recognition may include a complex cognitive ability involving recognition, memory, and an inner template or mental image of known individuals [31,32]. The mental processes involved in these cognitive abilities generally differ from those of simple innate releasing mechanisms [3,4]. In addition, we examined the individual recognition ability based on individual-specific facial features in three-spined sticklebacks, along with the significance of red nuptial colouration in aggressive male behaviour. The results of the present study will contribute to the resolution of questions regarding the cognitive abilities of this model animal.

## 2. Methods

### 2.1. Animals

The three-spined stickleback *G. aculeatus* is widely distributed north of 35° N, where it mainly inhabits coastal areas and the lower reaches of rivers [5,7]. During the breeding season, males establish a breeding territory containing a breeding nest and display a nuptial colouration consisting of a bluish dorsal surface and red abdomen [33,34]. The establishment of a territory precedes nesting [35,36], and males attack intruders in their territory by adopting a head-down posture and aggressively biting the fins of the intruders [4,33]. Males become more aggressive in defending their breeding territory during the breeding season than during other seasons; however, they have foraging territories in all seasons [37,38].

Fish were obtained from the sea-run population of the Shiomi River, Akkeshi, Hokkaido, Japan, bred at the World Freshwater Aquarium Aquatotto Gifu and kept in aerated stock tanks in a laboratory of Osaka City University. Fish were fed commercial flake food (TetraMin; Tetrawerke, Melle, Germany) and bloodworms (Chironomidae, *Propsilocerus akamusi*) once daily. Twenty fish consisting of 10 males (60–71 mm standard length [SL]) and 10 females (67–79 mm SL) were used in experiments conducted from August to November 2020 in experimental tanks of water at 15°C under a 12 : 12 h light/dark cycle. Owing to the constant room temperature and light/dark cycle, the nuptial colours of the males remained present in November, although the breeding season had ended in the wild. Fish behaviour was recorded using a video camera (HDR-CX470; Sony, Tokyo, Japan) in all experiments.

### 2.2. Procedure of experiment 1

**Figure 2.**
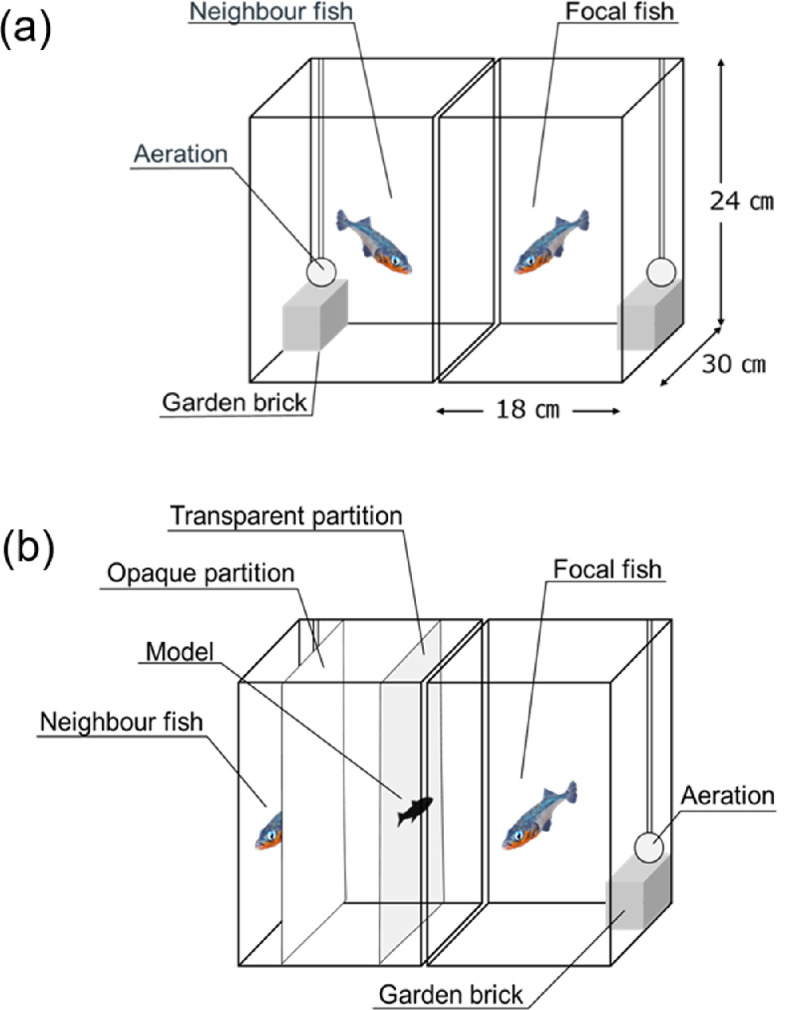
Tank layouts for experiment 1 (a) and experiment 2 (b). (a) One fish was introduced into each tank (18 cm × 30 cm × 24 cm). A gardening brick and an air stone were placed in each tank, and an opaque partition was removed before the initiation of the experiment. (b) A photo-model was set on the transparent partition after the neighbour fish was concealed by an opaque partition. Experiments began after the opaque sheet between the two tanks had been removed.

Familiar neighbours were created using an established procedure [26]. Fish were kept in isolation for more than one month in stock tanks, and two size-matched fish of the same sex (size difference <9 mm) were placed in adjacent tanks measuring 18 cm ×  30 cm × 24 cm (height) (*n* = 10 males or females in five combinations; each fish had a neighbouring fish of the same sex) (figure 1). Fish could only make visual contact with each other (figure 2). These experimental tanks contained an air stone and gardening brick (10 cm × 5 cm × 5 cm) to make the fish more aware of their territoriality.

Similar to previous studies using *Neolamprologus pulcher* [23,24,26], the period for establishing a dear enemy relationship between adjacent territorial individuals was set at 5−7 days. In our preliminary observations, three-spined sticklebacks also showed decreased aggressive behaviour towards adjacent territorial individuals and stabilized their aggression within 7 days, which then increased again when stranger individuals were presented (I. I. 2021, personal observation). Therefore, we recorded their behaviour on video for 30 min every 7 days. On day 1, we recorded the time immediately after the partition was removed (09.00), and on days 2−7, we recorded the time around noon. The time of aggressive behaviour of the focal fish towards neighbouring individuals was analysed for 5 min per day for 7 days. We measured the time taken to bite the glass wall facing the neighbouring tank with an open mouth, which is a common aggressive behaviour of three-spined sticklebacks against conspecifics [14]. The decrease in aggression against familiar neighbours, known as the dear enemy effect [19,20], indicates that animals can discriminate familiar neighbours from strangers or recognize individual familiar neighbours [26,31]. We placed an opaque partition between the two tanks after the lights were turned out on the final day of experiment 1.

### 2.3. Procedure of experiment 2

Experiment 2 was conducted after experiment 1, when the neighbours became less aggressive towards each other. In experiment 2, we acquired photographs of the side views of 10 familiar neighbours (N) and strangers (S) that had not been encountered by the focal fish for more than one month using a digital camera (Nikon D610; Nikon, Tokyo, Japan) with two lights (Digital Data Light DL-500, L18283). Because the red nuptial colouration of males was more distinct when they were face to face with other males, we acquired photographs before the end of experiment 1. These images were edited using GIMP 2.10.28 (The Gimp Team, https://www.gimp.org) to exchange parts of the face between familiar neighbours (N) and strangers (S).

**Figure 3.**
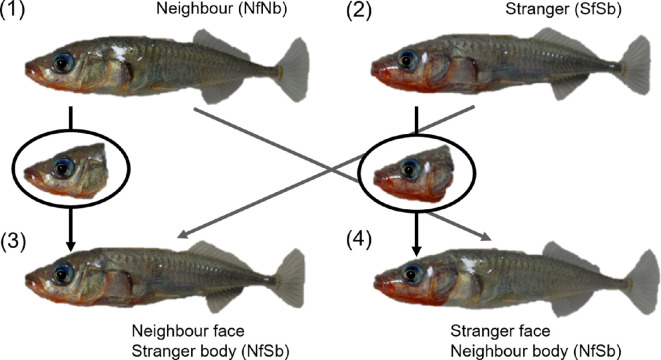
Photo-models presented in experiment 2. NfNb, familiar neighbour; NfSb, face of familiar neighbour on body of stranger; SfSb, stranger; SfNb, face of stranger on body of familiar neighbour.

For each focal fish (10 males and 10 females), we created 4 types of composite photographs with different face/body combinations: face and body of familiar neighbours (neighbour-face and neighbour-body: NfNb), face of familiar neighbour and body of stranger (NfSb), face and body of stranger (SfSb) and face of stranger and body of familiar neighbour (SfNb) (figure 3). We considered the facial area to include the operculum and defined the head as indicated in figure 3, similar to the creation of composite photographs of other fish species [22,24,26,28]. A slight adjustment of body colour tone was performed using GIMP to maintain consistent body colouration and to ensure that the margin between the visually transplanted face and body was not obvious to human observers (figure 3). We printed the four models on commercially available glossy photographic paper using an inkjet printer (EP-30VA; Epson, Nagano, Japan); the models were cut to match the contours of the fish and laminated.

We presented one of the four photo-models once every 2 days in random order for 5 min, 4 times in total over 8 days, at approximately 12.00 in all cases. During preliminary experiments, focal fish often fled from the photo-models attached to the glass wall and remained in the corner of the tank. Therefore, we placed a white opaque partition in the neighbouring tank to conceal the neighbour from the focal fish and placed a transparent partition with a photo-model 5 cm from the centre of the boundary of the focal fish tank (figure 2). After 5 min of acclimatization, when the focal fish resumed normal swimming, we gently removed the opaque partition between the two tanks and presented the photo-model for 5 min. During the presentation of the model, the behaviour of the focal fish was recorded on video.

We measured the duration of aggressive behaviour of the focal fish against the model for 2 min after the model had been presented, and the focal fish had found the photo-model. This period of observation was appropriate because of the gradual decrease in the behaviour of the focal fish against the motionless photographs during the presentation of the photo-model, which is consistent with a previous study [26].

Because the focal fish were more aggressive towards strangers than familiar neighbours (see §3), we predicted that the time spent on aggressive behaviour would be longer in the stranger model (SfSb) than in the familiar neighbour model (NfNb). If three-spined sticklebacks recognized familiar conspecifics only by their facial features, their responses to models with the neighbour-face (NfNb and NfSb) would be similar regardless of body, and their responses to models of the stranger-face (SfSb and SfNb) would also be similar regardless of body. In contrast, if these fish used the whole body or features of both the face and body to recognise conspecifics, their responses to the modified model (NfSb and SfNb) would presumably be intermediate in comparison to their responses to the unmodified model (SfSb, NfNb).

The size of the red area varied considerably among the male fish (figure 1a,b). Therefore, we measured the area of nuptial red colour between the snout and base of the pelvic fin in the photographs using Adobe Photoshop 23.1.1. (Adobe Systems Inc., San Jose, CA, USA). We then converted the photographs to black and white binary data using ImageJ (http://rsb.info.nih.gov/ij/) and calculated the ratio of the red area to the body area, excluding the fins. If the red area plays a role in triggering male aggression, focal fish would be expected to show greater aggression towards photo-models with greater nuptial colour.

### 2.4. Statistical analysis

Statistical analyses were performed using R v. 4.3.2 (R Core Team, 2023) [39]. Linear mixed models (LMMs) in the R packages *lme4* and *lmerTest* were used for all analyses, with individual ID as a random effect to handle the repeated-measures design of the within-individual observation. All LMMs were fitted using restricted maximum likelihood, and *F*-tests with Satterthwaite’s method were applied to test statistical significance. The effect sizes (partial *η^2^*) and their 95% confidence intervals (CI) were calculated using *effectsize* in the R package. For post hoc comparisons, Tukey’s contrasts were used with the *glht* function in the R package *multicomp*. Two-sided *p-*values <0.05 were considered statistically significant for all tests.

In experiment 1, we assessed whether the time spent being aggressive by focal fish against neighbours of the same sex decreased from day 1 to day 7 by constructing a separate LMM for each sex (*n* = 10 males and 10 females). In experiment 2, we compared the time spent being aggressive by focal fish directed towards four photographic models of the same sex (NfNb, NfSb, SfSb and SfNb) in a separate LMM for each sex (*n* = 10 males and 10 females). Finally, we tested whether sign stimuli affected the aggressive behaviour of focal males against photographic models of the same sex. We constructed an LMM with the time spent being aggressive by focal males (*n* = 10) against photographic models as a response variable, a photographic model (two models: NfNb and SfSb) as the fixed factor and the ratio of the red area to the total body area as the covariate, including their interaction.

## 3. Results

### 3.1. Experiment 1

**Figure 4.**
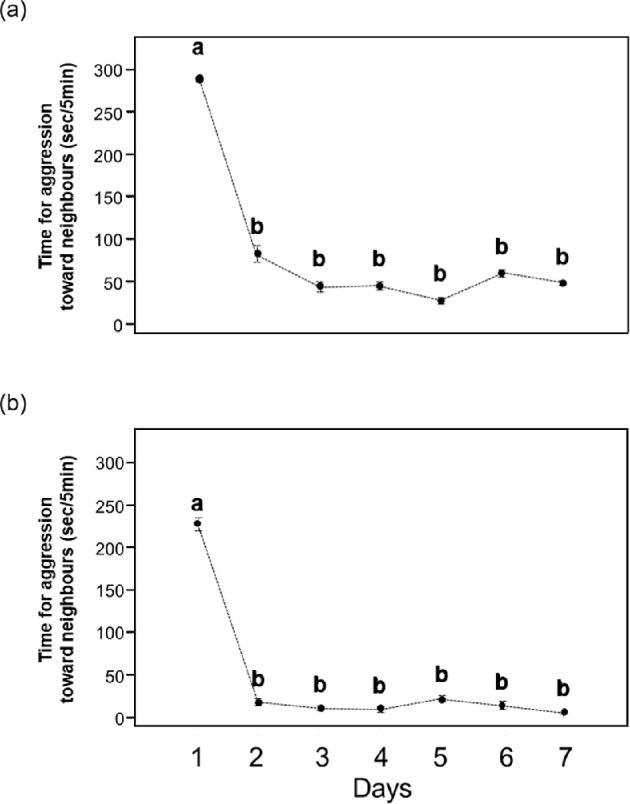
Changes in the time focal males (a) and females (b) spent acting aggressively towards neighbours over a 7-day period. Means ± s.e.m. (sec/5 min; *n* = 10 for each sex). Different alphabets denote statistically significant differences (a versus b, *p* < 0.05) by linear mixed models (LMMs) followed by Tukey contrasts.

The time spent in aggression by focal fish against neighbours of the same sex was highest immediately after the opening of the partition on day 1 (male: 290.48 s ± 4.56 s.e.m. per 5 min, *n* = 10, female: 227.84 s ± 15.80 s.e.m. per 5 min, *n* = 10; figure 4). On day 2 and subsequent days, the time spent in aggression by focal fish decreased significantly in both sexes (male: LMM, *F*_6,54_ = 105.69, *p* < 0.0001, partial *η^2^* = 0.92 [95% CI = 0.89–1.00]; figure 4a; female: LMM, *F*_6,54_ = 80.38, *p* < 0.0001, partial *η^2^* = 0.90 [95% CI = 0.86–1.00]; figure 4b) and stabilized at a lower level (male: < 80 s per 5 min; female: < 25 s per 5 min).

### 3.2. Experiment 2

**Figure 5.**
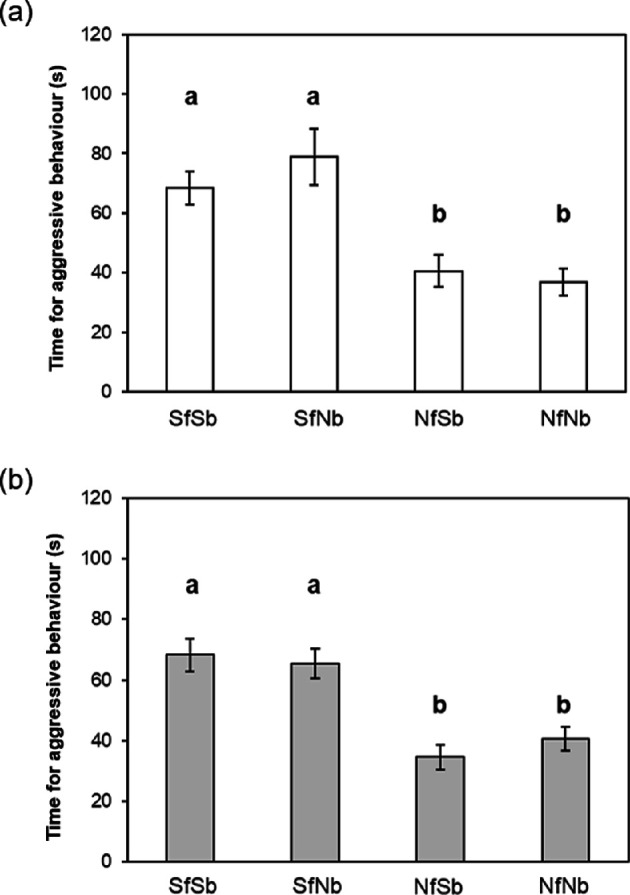
Times spent in aggressive behaviour by focal fish against photo-models recorded in experiment 2. Males (*n* = 10) (a) and females (*n* = 10) (b). NfNb, familiar neighbour; NfSb, face of familiar neighbour on body of stranger; SfSb, stranger; SfNb, face of stranger on body of familiar neighbour. Means ± s.e.m. a versus b: *p* < 0.05 by linear mixed models (LMMs) followed by Tukey contrasts.

Focal fish of both sexes exhibited aggression differently towards four photo-models of the same sex (male: LMM, *F*_3,36_ = 12.35, *p* < 0.0001, partial *η*^2^ = 0.51 [95% CI = 0.29–1.00]; figure 5a; female: LMM, *F*_3,36_ = 13.59, *p* < 0.0001, partial *η*^2^ = 0.53 [95% CI = 0.32–1.00]; figure 5b). As predicted, focal fish of both sexes spent more time being aggressive towards stranger (SfSb) photo-models than towards familiar neighbour photographic models (NfNb). Furthermore, regardless of the body, focal fish aggression was significantly greater toward both stranger-face photographic models (SfNb and SfSb) than towards both neighbour face photographic models (NfSb and NfNb) (figure 5).

Additionally, the ratio of the red area on the face to belly to total body area of male fish had no effect on the time spent in aggression by focal males (LMM, photographic model × nuptial colour: *F*_1,16_ = 0.22, *p* = 0.65, partial *η*^2^ = 0.01 [95% CI = 0.00–1.00]; nuptial colour, *F*_1,16_ = 0.05, *p* = 0.83, partial *η*^2^ = 0.003 [95% CI = 0.00–1.00]; photographic model; *F*_1,16_ = 12.39, *p* = 0.003, partial *η*^2^ = 0.44 [95% CI = 0.13–1.00]).

## 4. Discussion

The results of the present study suggest that three-spined sticklebacks of both sexes show dear enemy relationships with their neighbours and considerably reduced aggressiveness towards familiar neighbours. The aggression frequency was much lower against photo-models of familiar faces than against models of stranger faces, independent of body type, clearly indicating that three-spined sticklebacks can distinguish familiar neighbours from strangers via facial recognition. Individual recognition based on facial features has been previously reported in fish belonging to the orders Perciformes [23,25,27,29], Beloniformes [30] and Cyprinodontiformes [27]. The three-spined stickleback belongs to the order Gasterosteiformes. These four fish orders are not closely related [40], suggesting that familiar recognition by facial recognition is widespread among teleosts.

In territorial species, a stranger represents a high level of threat to territory owners, whereas a familiar dear neighbour rarely intrudes across the territorial boundary, representing a low level of threat (in birds e.g. [18,19]; in fish [16,21–23]). Therefore, the current results suggest that the three-spined stickleback changes aggression levels according to the threat level of adjacent individuals. This suggestion is consistent with previous studies showing that stickleback aggression is affected by the location of rival males [36]. Rapid identification of individuals around an individual’s territory is indispensable for the effective maintenance of a territory through dear enemy relationships [33]. Familiar recognition of dear neighbours has been reported in birds (e.g. [18,20] and territorial cichlid fishes [23,25,26]); however, these examples solely involved males. Male three-spined sticklebacks have breeding territories, and female three-spined sticklebacks have feeding territories [31] and familiar recognition of both sexes plays an important role in their social lives.

Why do sticklebacks and other fish, including cichlids, guppies and medaka, distinguish familiar fish based on facial features rather than on other body parts? Eyes play an important role in facial recognition in animals and humans (e.g. fish [41,42], primates [43,44] and humans [45–47]). Studies on eye tracking in primates and humans have shown that they initially tend to focus on an opponent’s face, especially the eyes, before focusing on other body parts (e.g. humans [46], chimpanzees [47,48] and rhesus monkeys [44]). This suggests that the visual cues of individual signals located near the eyes allow rapid signal transmission in these species. Recently, it has been reported that the cichlid *N. pulcher* gazes at the face of the encountered fish first and for significantly longer than at other body parts [47], which is consistent with facial recognition patterns in mammals (e.g. [45–47]) and birds [49]. Notably, *N. pulcher* can distinguish between familiar and unfamiliar faces within 0.5 s with a high accuracy [23]. For this type of rapid and accurate facial recognition, individual-specific signals near the eye (i.e. individual variations in face colour morphs [figure 1a,b]) should also be effective in the three-spined stickleback.

Three-spined sticklebacks can discriminate between familiar neighbours and strangers based on individual faces. Facial features represent individual-specific signals, and this facial recognition process is similar to true individual recognition (TIR) rather than class-level recognition [25,31,50]. The cichlid *N. pulcher*, which can distinguish familiar dear neighbours from strangers, can identify two familiar dear neighbours based on their specific facial features (i.e. TIR) [23,26]. We predicted that the three-spined stickleback, a species with individually specific faces, could also perform TIR [32]. The ability to recognize multiple dear neighbour fish (i.e. TIR) is necessary to effectively maintain stable territoriality. This cognitive ability is likely to be highly sophisticated, similar to that of mammals [32]. Our observations suggest that sticklebacks that can recognize and respond appropriately to a familiar individual will have a complex cognitive capacity, which could be associated with mental mechanisms different from the innate releasingmechanism with a sign-stimulus.

However, in the present study, we observed cases in which four males lacking the red colour received intensive aggression on day 1 in experiment 1; the size of the red area, including its absence, had no effect on the intensity of male aggression in experiment 2 (figure 1b). This is consistent with previous reports that the degree of red nuptial colouration of the belly of this fish has no effect on the intensity of territorial aggression [10,11]. However, it should be noted that because the models presented here were photographs, it is not possible to comment on the effect of chemical stimuli and the probability of winning or the intimidating effect that the red belly might influence [12]. Furthermore, females lacking the red colour exhibited intense aggression against unfamiliar females. In any case, our observations do not support the interpretation of aggression prompted by the ‘sign-stimulus’

Milinski [13] reported that three-spined sticklebacks use a tit-for-tat strategy during repeated predator inspection visits. Such a strategy requires the TIR of opponents and a strong memory [51]. The results of the present study provide evidence for familiar recognition and suggest the presence of TIR in sticklebacks. A recent study showed no evidence of familiar recognition in the three-spined stickleback [15]; however, a lack of evidence of capacity does not always constitute evidence of its absence. Perhaps, considering the present results and the ecology of sticklebacks, they are able to associate rocks and food; however, no relationship was found between recognized individuals and food. Because associating rocks and food is part of their daily life, but associating individuals and food is rare. Many examples of high cognitive abilities in social fishes have been reported thus far, including the prediction of the behaviour of others based on an individual’s own experience during coordinated hunting [52], transitive inference of social dominance [53,54], the use of prosocial and antisocial choices [55] and mirror self-recognition [56–58]. These sophisticated forms of social cognition and maintenance of stable sociality, such as dominance order and territoriality, require the ability to recognize specific individuals. The present findings suggest that three-spined sticklebacks represent the evolution of some intelligence or cognitive abilities that are not explained as responses to sign stimuli in fish within the context of complex social relationships [59].

## Data Availability

Supplementary material is available online [60].
